# Ecological and Biological Properties of *Satureja cuneifolia* Ten. and *Thymus spinulosus* Ten.: Two Wild Officinal Species of Conservation Concern in Apulia (Italy). A Preliminary Survey

**DOI:** 10.3390/plants10091952

**Published:** 2021-09-18

**Authors:** Enrico V. Perrino, Francesca Valerio, Shaima Jallali, Antonio Trani, Giuseppe N. Mezzapesa

**Affiliations:** 1 CIHEAM, Mediterranean Agronomic Institute of Bari, Via Ceglie 9, 70010 Valenzano, Italy; jellelishaima@yahoo.com (S.J.); trani@iamb.it (A.T.); mezzapesa@iamb.it (G.N.M.); 2 National Research Council of Italy, Institute of Sciences of Food Production, Via Amendola 122/O, 70126 Bari, Italy; francesca.valerio@ispa.cnr.it

**Keywords:** correlation, ecology, essential oils, Lamiaceae, vegetation

## Abstract

This study evaluated the effects of ecology (plant community, topography and pedology), as well as of climate, on the composition of essential oils (EOs) from two officinal wild plant species (Lamiales) from Apulia, namely *Satureja cuneifolia* Ten. and *Thymus spinulosus* Ten. Few scientific data on their chemical composition are available, due to the fact that the first has a limited distribution range and the second is endemic of southern Italy. Results for both species, never officially used in traditional medicine and/or as spices, showed that the ecological context (from a phytosociological and ecological point of view) may influence their EO composition, and hence, yield chemotypes different from those reported in the literature. *S. cuneifolia* and *Th. spinulosus* can be considered good sources of phytochemicals as natural agents in organic agriculture due to the presence of *thymol* and *α-pinene*. Overall, the obtained trend for EOs suggests a potential use of both species as food, pharmacy, cosmetics and perfumery. Hence, their cultivation and use represent a positive step to reduce the use of synthetic chemicals and to meet the increasing demand for natural and healthier products.

## 1. Introduction

Medicinal and aromatic plants (MAPs) have a long history of being used for multiple purposes, including for food and therapeutics. Before the advances of modern medicine, ancient civilizations discovered a wealth of useful therapeutic agents from within the plant and fungi kingdoms [1]. Knowledge of these medicinal preparations was passed down through generations and was occasionally recorded in the herbal literature [2]. Hence, the use of these wild officinal plants became a significant aspect of populations’ cultural heritage and transformed into different traditions that kept on being passed down [3,4]. Much research in the field of biology, chemistry and medicine is directed at the identification and characterization of plant secondary metabolites with a pharmacological activity, which may be candidates for the synthesis of new drugs [5].

MAPs are defined as plants possessing aromatic and/or medicinal characteristics that can be extracted in the form of essential oils (EOs) through their secondary metabolites of bioactive properties [6]. These extracts are synthesized for several reasons, including plant protection against pathogens, insects, pollinators attraction and for allelopathic activity [7,8,9].

EOs are natural complexes of volatile and semi-volatile organic compounds, responsible for specific aroma, flavor and fragrance of certain plant species [10]. They can be stored by all plant organs (flowers, leaves, stems, twigs, seeds, fruits, roots, wood, or bark) and are synthesized in different histological structures such as secretory cells, cavities, canals or glandular trichomes [8,11].

The chemical structure of plant EOs falls into two distinct classes: terpenes and phenylpropanoids. Terpenes are formed by the combination of isoprene units such as monoterpenes (two units), sesquiterpene (three units), diterpenes (four units), and phenylpropanoids, which are aromatic and non-terpenoid compounds responsible in part for the fragrance of the plant [11,12]. These volatile oils represent a wide chemical diversity and are responsible for many plant applications, such as antimicrobial, antiparasitic, insecticidal, herbicidal, antioxidant and other medicinal properties.

The Lamiaceae family (formerly known as Labiatae) is the largest family of the order Lamiales, containing more than 245 genera and 7886 species [13], which grow in different natural ecosystems worldwide, with a particularly high concentration in the Mediterranean region, including Italy, providing a great level of biodiversity [14]. Most of the species belonging to this family are aromatic and possess EOs, making them valuable in cosmetics, perfumery, agriculture and medicine [15,16].

EOs can present a wide variability in their yield and chemical composition due to a series of intrinsic and extrinsic factors that affect the plants in their environment. In fact, it is well documented that the same species, under different environmental and geographical conditions, can produce EOs with different chemical profiles and biological properties [17,18,19]. In addition, some of these taxa are rare or endemic and have not yet been investigated for their ecology, chemical composition and biological properties.

The aim of the present research was to investigate two wild species of conservation concern (SCC; species for which we have concerns about its ability to remain on a landscape for a long time), *Satureja cuneifolia* Ten. (wild savory) and *Thymus spinulosus* Ten. (little thorn thyme), in order to: (a) perform ecological studies to understand the relationships existing between plant associations (sociology) and their surrounding habitat; (b) verify and eventually explain the ecological context influences on the composition of EOs; (c) determine chemical composition; (d) evaluate the potential for commercial purpose.

## 2. Study Area

**Figure 1:**
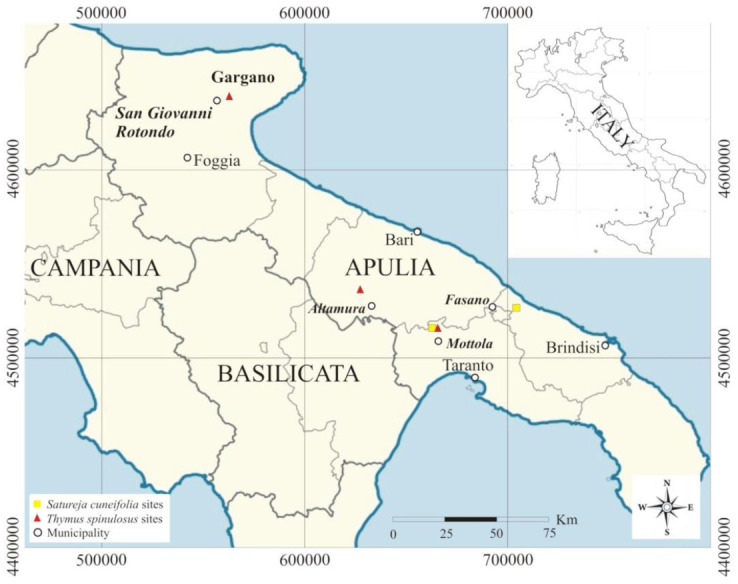
Site locations of *Satureja cuneifolia* and *Thymus spinulosus*.

Samplings were carried out in 2020 in Apulia. For *Satureja cuneifolia*, two sites, San Basilio (Mottola, province of Taranto) and Difesa di Malta (Fasano, province of Bari), were detected, while for *Thymus spinulosus*, three sites were identified, one of which, San Basilio (Mottola), was the same as *S. cuneifolia*, and the other two sites being Scannapecora (Altamura, province of Bari) and S. Egidio in S. Giovanni Rotondo (province of Foggia) (Figure 1).

The investigated sites have a Mediterranean macro-bioclimate, except S. Egidio, which is cooler, resulting Temperate. The bioclimate is oceanic, particularly the semicontinental transition at S. Egidio and Scannapecora, while the ombrotype is always the subhumid and dry type at Difesa di Malta (Table 1) (Phytoclimatic map—Italian Ministry for the Environment, Land and Sea, http://www.pcn.minambiente.it/viewer, accessed on 26 May 2021) [20] (Table 1).

Within the San Basilio site in the province of Taranto, the most widespread pedotype is Lithic Ruptic–Inceptic Haploxeralf fine, predominantly clayey, very thin, and very rocky with substrate within 50 cm [21]. At Scannapecora in the province of Bari, the geological type is that of Skeletal limestones of neritic and carbonate platform facies (Upper Cretaceous), on Tyrrhenian carbonate reliefs with material defined by calcareous sedimentary rocks and climate from Oceanic to Suboceanic Mediterranean, partially mountainous. The Difesa di Malta and S. Egidio sites share the same ecopedology as the Scannapecora site but different geopedology and geolithology, respectively, with Terrigenous-skeletal limestones such as “Panchina” (Pleistocene) and Colluvial, terraced alluvial, fluviolacustrine and fluvioglacial deposits (Pleistocene) (Geological, Geolithological and Ecopedologic maps—Italian Ministry for the Environment, Land and Sea, http://www.pcn.minambiente.it/viewer, accessed on 26 May 2021) [20] (Table 2).

## 3. Materials and Methods

### 3.1. Vegetation Analysis

The field inspections of *Satureja cuneifolia* and *Thymus spinulosus* were carried out in 2020. A total of five vegetation surveys were conducted for both species, following the phytosociological method of the Zurich–Montpellier Sigmatist School [22], updated by Géhu and Rivas-Martínez [23], using the coefficient of abundance–dominance of identified species, well known to field botanists, and concerning the covering percentage of the relief area (value 5: species with more 75% coverage; value 4: species with coverage from 50% to 75%; value 3: species with coverage from 25% to 50%, value 2: species with coverage from 5% to 25%: value 1: species with coverage from 1% to 5%; value +: species with coverage less than 1%). Identification code, taxon, location, date, and geographical position (expressed in WGS84—World Geodetic System 1984) are reported in Table 3, while phytosociological and topographical data (identification code, altitude (m a. s.), aspect, slope (°), relevé area (m^2^), stoniness (%), rockiness %), cover total (%), soil depth detected (cm), macroclimate, geolithological and eco-pedological types), endemicity, number of species identified, and number of individuals collected for laboratory analyses are reported in Table 4 and Table 5. Other identified and recorded species not used for the phytosociological classification are not reported here. A total of 5 specimens for each species and for each reléve were collected and deposited at the official herbarium of Bari University (Italy) (*Herbarium Horti Botanici Barensis*—BI).

Identification of the taxa was carried out according to Flora d’Italia [24] and Flora Europea [25], with nomenclature standardized by “An updated checklist of the vascular flora native to Italy” [26] and “An updated checklist of the vascular flora alien to Italy” [27], while the syntaxonomic framework was conceived by several contributions [28,29,30], reported in the phytosociological tables and summarized in the syntaxonomic scheme.

The aerial parts of the studied plant species were harvested in the same sites and date of vegetation surveys. The number of individuals collected (from 15 to 100) for the laboratory analysis is reported in the phytosociological tables (Table 4 and Table 5).

### 3.2. Essential Oils Analysis

The essential oils (EOs) of the two wild plant species were extracted by hydrodistillation [31] using a Clevenger type apparatus for 4–5 h. Air-dried plant material (100 g) was covered with 500 mL of distilled water in a 1L volume distillation flask (extraction ratio 1:5 w/vol). EOs were collected in amber glass vials, weighed, and stored at 4 °C. EO yields (% *w*/*w*) were determined as grams of EOs per 100 g of dry weight plant material.

Identification of the compounds present in the EO extracts was carried out by gas chromatography coupled with mass spectrometry (GC–MS) using a Clarus 680 GC interfaced with a Clarus SQ8C single quadrupole mass spectrometer (PerkinElmer, Waltham, MA, USA) equipped with an Elite-5 MS (PerkinElmer, Waltham, MA, USA) fused silica capillary column (30 m × 0.25 mm and 0.25 μm film thickness). Mass spectra of the target compounds were obtained by an electron impact ionization system with standardized ionization energy of 70 e V. Helium 5.5 was used as carrier gas at a constant flow rate of 1ml/min. Mass transfer line and injector temperatures were set at 280 °C and the oven temperature was programmed from 50 °C to 160 °C at 5 °C/min, then raised to 250 °C at 10° C/min, and held at the final temperature for 5 min. Diluted samples (1:10, *v*/*v*, in hexane) were injected in split mode with a split ration of 1:100. Data were collected in full scan mode in the range 40−300 amu. A solvent delay of 4 min was applied. Qualitative results include compound identification and area percentage of related peaks in the total ions chromatogram (Table 5).

Compounds identification was performed by both Kovats retention indexes (RI) [32,33] and mass spectra (MS) searches in NIST and Wiley databases (Table 6).

The chemical composition (%) of the main compounds of *Satureja cuneifolia* and *Thymus spinulosus* is reported in Table 7. Statistical analysis was not applicable due to the low number of samples per species and site, limited amount of material per sample, and few and variable numbers of individuals per specimen (15–70), also due in part to the fact that the study was carried out on two species of conservation concern, which limits the harvest of material.

### 3.3. Soil Analysis

Soil samples were collected from each vegetation sample of topsoil in the following way: in 5–9, subsamples (depending on the habitat extension; each subsample ∼ 100 g) were taken from the first 0–20 cm of depth.

Soil samples were sieved to <2 mm and stored for physical and chemical analysis. Particle size distribution was determined by the pipette method, whereas textural class was categorized according to the USDA classification [34]. Soil pH was measured both in water and saline solution using 1:2.5 ratio (*w/v*). Electrical conductivity (EC) was measured on a soil to distilled water ratio (1:2, *w/v*). Soil organic carbon (OC) was determined according to the Walkley and Black method as described by Nelson and Sommers [35]. Total nitrogen (N) was measured following the Kjeldahl method as described in Bremner [36]. Available P was measured in sodium bicarbonate alkaline soil extracts and determined colorimetrically [37]. The total carbonate content in the soils was determined using a Dietrich–Fruhling calcimeter. Exchangeable bases were extracted by BaCl_2_ (1 M) and triethanolamine solution, while available microelements were extracted by DTPA and triethanolamine solution. After extractions, both were analyzed by ICP-OES.

## 4. Results and Discussions

### 4.1. Satureja Cuneifolia Ten.

**Figure 2:**
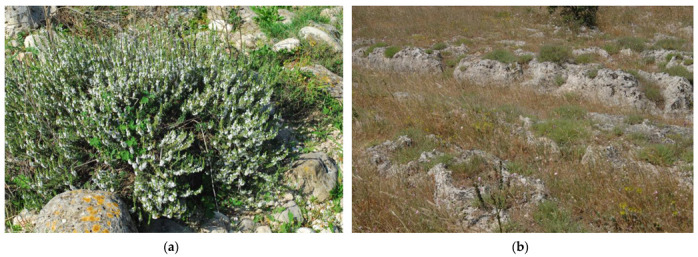
*S. cuneifolia* (**a**) in flowering; (**b**) in its habitat, *Phagnalo saxatilii-Saturejetum cuneifoliae*. San Basilio, 26 March 2020.

*S. cuneifolia* (synonym: *Satureja montana* subsp. *cuneifolia* (Ten.) O. Bolós & Vigo) is shown in Figure 2.

**Figure 3:**
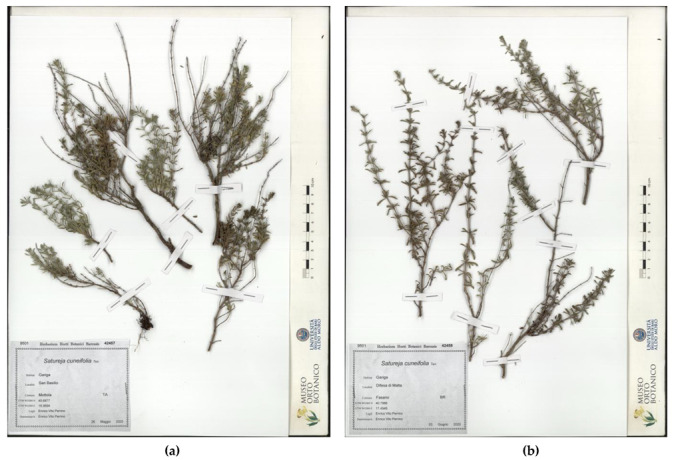
*S. cuneifolia* herbarium samples: (**a**) San Basilio (Mottola) (BI 42457); (**b**) Difesa di Malta (Fasano) (BI 42458).

*Herbarium* samples are shown in Figure 3: The sample from San Basilio, in the municipality of Mottola (Taranto), garrigue, 26 May 2020, *legit* and *determinavit* determinative *E.V. Perrino* 42457 (*Herbarium Horti Botanici Barensis*—BI), is shown in Figure 3a. The sample from Difesa di Malta, in the municipality of Fasano (Brindisi), garrigue, 3 June 2020, *legit* and *determinavit E.V. Perrino* 42458 (*Herbarium Horti Botanici Barensis*—BI), is shown in Figure 3b.

The genus *Satureja* L. comes from the Latin “*satureia*” and was first named by Roman writer Pliny [38]. It means “*herb of satyrs*” and, for this reason, its cultivation was banned in monasteries [39]. *S.* belongs to the Lamiaceae family, sub-family *Nepetoideae*, and the tribe *Mentheae* [40] including about 200 wild species of herbs and shrubs, often aromatic, widely distributed in the Mediterranean area, Asia and boreal America [41,42]. More than 30 of them grow in eastern parts of the Mediterranean area [43,44], up to 1200 m above sea level, in arid, sunny, stony and rocky habitats [45], 5 of which are in Italy: *S. cuneifolia* Ten., *S.* *montana* L. subsp. *montana*, *S. montana* L. subsp. *variegata* (Host) P.W. Ball, *S. subspicata* Bartl. ex Vis. subsp. *liburnica* Šilic, and *S. thymbra* L. [26]. *S. cuneifolia* Ten. is a SE European entity reported in Italy, Albania, Greece, Croatia and Montenegro [46,47], with its western limit of distribution in Italy, where the species occurs only in Apulia, throughout the Region, in Salento, Murge and Gargano [48,49,50,51,52], and in the nearby Basilicata, in the Murge di Matera [53]; it was recorded by mistake at Laino Borgo in Calabria [24,26]. As highlighted by some authors [54], this taxon is one of the many plant species with eastern distribution that exhibit a disjunct Italian distribution more or less restricted to the Apulian region, such as *Bromus parvispiculatus* H. Scholz [55], *Carex phyllostachys* C. A. Mey. [56,57], *Cerinthe retorta* Sm. [58], *Linum elegans* Spruner ex Boiss. [59], *Scrophularia lucida* L. [60,61], and *Ophrys oestrifera* complex [62].

*S. cuneifolia* grows from sea level to 600 m of altitude, and is a diagnostic taxon of three plant communities: (a) *Phagnalo saxatilii-Saturejetum cuneifoliae* Biondi & Guerra (2008) associated with *Phagnalon saxatile* (L.) Cass.; (b) *Sedo ochroleuci-Saturejetum cuneifoliae* Di Pietro & Misano (2010) associated with the endemism *Petrosedum ochroleucum* (Chaix) Niederle subsp. *mediterraneum* (L. Gallo) Niederle; (c) *Stipo austroitalicae-Seslerietum juncifoliae* Di Pietro & Wagensommer (2014) subass. *typicum* [54]. These vegetations fall into the *Ononido-Rosmarinetea* class, with the first two (a, b) described for central of Apulia in the “Gravine” gorges [49,63], while the third (c) for the Gargano [50,54]. The *Stipo austroitalicae-Seslerietum juncifoliae* subass. *typicum* grows on small rocky outcrops emerge from a matrix composed of limestone debris, found within the steep slopes of the gorges, which cut across the southern side of the Gargano promontory [54]; it could be considered a distinct aspect of the habitat “Calcareous rocky slopes with chasmophytic vegetation” (code 8210) according to Directive 92/43 EEC [64,65]. 

*Satureja* species have been traditionally used in the treatment of many diseases such as nausea, indigestion, cramps, diarrhea, infectious diseases and muscle pains [66,67], in relation to the EOs secreted by the glands found on their leaf surface, as with other aromatic plants belonging to some genera of the Lamiaceae family.

Previous studies from the Mediterranean area showed variability in chemical composition in EOs of *S. cuneifolia*, attributed to many factors such as climate, regional, local and ecological conditions, growth stages, harvesting period, and genetics [67,68,69,70,71]. In three different localities of Dalmatia (Croatia), corresponding to as many stages of development, the yield of the EOs ranged from 0.1% to 0.6%, and all samples had a low percentage of thymol (0–3.5%) and carvacrol (0–16.3%), but were relatively rich in α-pinene (1.3–20.7%), limonene (1.8–17.4%), γ-terpinene (0–5.6%), and p-cymene (1.8–14.8%) [69]. In a research study carried out in the southwestern part of Montenegro (Lovćen National Park), a high percentage of linalool (20.3%) has been found, the most abundant compound, followed by much lower values of α-terpineol (3.8%) and borneol (3.6%), while limonene (1.1%) and α-pinene (0.7%) were under-represented, and carvacrol and thymol were even absent [67]. In high altitude conditions and precisely from Sogut mountain in Turkey, *S. cuneifolia* collected during the flowering stage in August recorded elevated values of carvacrol (45%), p-cymene (21.6%), and then, lower but appreciable values thymol (9%), γ-terpinene (4.3%), and borneol (2.5%) [72]. A further study [70] conducted in 12 wild sites far east of the Italian peninsula at lower altitudes than Turkey showed significant percentage changes in the main compounds observed: α-pinene (0.3–26.4%), linalool (0.2–45.8%) and borneol (1.2–41.1%), with linalool and α-pinene not always present. An accurate reading of the Italian data also highlights that linalool and α-pinene reached high values (>35% and >14%, respectively) only in four samples, while borneol was the most present chemotype.

*S. cuneifolia* from Croatia and Montenegro is very different from that collected in the Mediterranean parts of Turkey and eastern Italy (Apulia). EOs of Croatian and Montenegro origin have a low content (even absent) of both thymol and carvacrol, as observed in Italian sites, while, in general, EOs of plants from Turkey have a mean percentage of thymol accompanied by a high percentage of carvacrol or vice versa. In Italy, the values are even more extreme and variable because not only have thymol and carvacrol not been recorded, but they have considerable fluctuations within the same compound in comparable environments. The authors [70] explain that this intraspecific polymorphism indicates that homogeneous environments may host different genotypes.

The data collected about *S. cuneifolia* show that the vegetation belongs to *Phagnalo saxatilii-Saturejetum cuneifoliae* at both investigated sites (Table 4), where there is the same Mediterranean macroclimate, but different bioclimate and ombrotype, respectively: Dry Mediterranean Oceanic under the influence of the sea at Difesa di Malta, and Subhumid Oceanic in the hinterland at San Basilio. In addition to the climatic differences, there are also geolithological and ecopedological differences, with a more recent Pleistocene geology near the sea, where we find Terrigenous-skeletal limestones such as “Panchina”, and older in the hinterland (Upper Cretaceous) with skeletal limestones of neritic and carbonate platform facies. Differences were confirmed by Hilly reliefs with undifferentiated tertiary sedimentary rocks at Difesa di Malta and Tyrrhenian carbonate reliefs with material defined by calcareous sedimentary rocks at San Basilio. This means that the two sites only have the same macroclimate, but different bioclimate, geology, and pedology characteristics, and partially different plant communities. In fact, within the same plant association, we observe less human-made interference in San Basilio than Difesa di Malta, and a greater biodiversity due to the many species referable to the *Hippocrepido glaucae-Stipion austroitalicae* alliance and *Scorzoneretalia villosae* order, enhanced with two endemic taxa: *Scorzonera villosa* subsp. *columnae* and *Thymus spinulosus* Ten. The vegetation detected in San Basilio deserves more analysis from a syntaxonomic point of view, through ad hoc surveys in the surrounding areas, to better define this distinct aspect with *S. cuneifolia*.

The soil parameters show some differences between the two sites (Sc1, Sc2). In the more disturbed aspect, less natural, in a cultivated context, as Difesa di Malta (Sc1), a fine-silt loam soil has been recorded, characterized by poor total carbonate, very high phosphorus availability and high total nitrogen and organic carbon, while at San Basilio (Sc2), in a more sandy soil, lower and moderate values were recorded, except for the total carbonate which, here, has a much higher concentration (Table 5), confirming the geopedological and geolithological features (Table 4).

The exchangeable cation and available microelement (macro and micronutrients, respectively) soil contents showed that all samples are rich in exchangeable cation but less rich in available microelements, with differences in types and percentage at both sites. The available microelements have comparable concentrations at both sites, except for Zn and Mn, which are more abundant in natural contexts, unlike the Fe that prevails in agricultural land (Table 5). 

The environmental, geological, ecological, vegetational and pedological differences have a slight effect on the phytochemical properties of *S. cuneifolia*, remembering that the San Basilio site is located at a higher altitude (274 m), has a major plant biodiversity, and older soils, compared to the maritime site of Difesa di Malta (49 m), which is situated in a more agricultural context and in a more recent soil. In particular, the correlation between EO compositions was meaningful and positive with the monoterpenoid alkene and monoterpenoid alkene alcohol, which are the most abundant classes in *S. cuneifolia* EOs. Additionally, monoterpenoid alkenes have a significant positive correlation with soil pH, and significant negative correlation with total nitrogen and soil organic carbon (Table 5).

In all, 36 compounds were identified at the Difesa di Malta (Sc1) and San Basilio (Sc2) sites, but with some quantitative differences (Table 6). Most of the compounds are present with similar percentages at both sites. In particular, α-Pinene, the most abundant phytochemical, is present with the highest percentage at both sites (36.8% and 38.8%, respectively), the same slight difference occurs for limonene (5.1% and 6.4%), but significant differences arise for α-Terpineol (11% and 17.1%), trans-3-caren-2-ol (4.1% and 1.9%), and Borneol (6.9% and 4.1%). The rest of the compounds for both sites did not exceed 5%. 

Finally, even other minor compounds, not mentioned, were present with slightly different percentages.

### 4.2. Thymus Spinulosus Ten.

**Figure 4:**
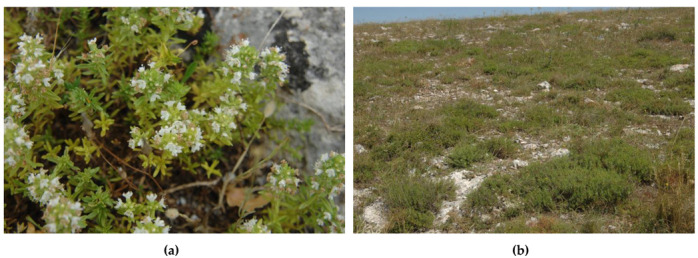
*Th. spinulosus* (**a**) in flowering; (**b**) in its habitat, *Hippocrepido glaucae-Stipion austroitalicae*. Scannapecora, 03.06.2020.

*Th. spinulosus* (synonym: *Th. paronychioides* Celak.) is shown in Figure 4. 

**Figure 5:**
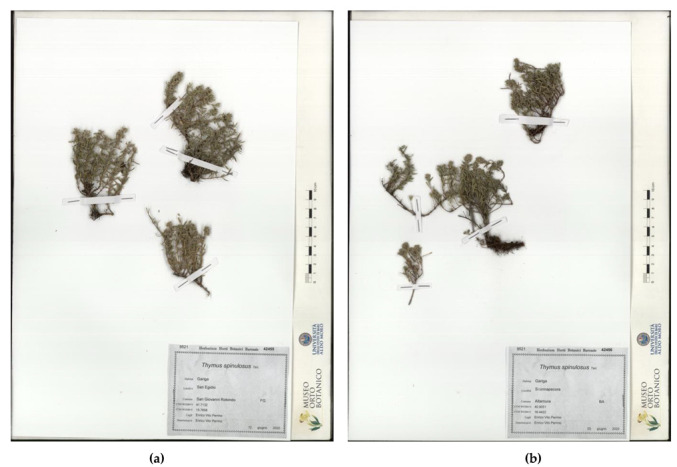
*Th. spinulosus* herbarium samples: (**a**) San Egidio (S. G. Rotondo) (BI 42455); (**b**) Scannapecora (Altamura) (BI 42456).

*Herbarium* samples are shown in Figure 5: The samples from San Basilio, in the municipality of Mottola (Taranto) (garrigue, 5 June 2020, *legit* and *determinavit E.V. Perrino* 42454 (*Herbarium Horti Botanici Barensis*—BI) and San Egidio, in the municipality of San G. Rotondo (Foggia), garrigue, 12 June 2020, *legit* and *determinavit* *E.V. Perrino* 42455 (*Herbarium Horti Botanici Barensis*—BI) are shown in Figure 5a. The samples from Scannapecora, in the municipality of Altamura (Bari), garrigue, 3 June 2020, *legit* and *determinavit E.V. Perrino* 42456 (*Herbarium Horti Botanici Barensis*—BI) are shown in Figure 5b.

The genus *Thymus*, described by Carl Linnaeus in *Species Plantarum* [73], is one of the most important genera within the family Lamiaceae, due to its high number of species, commercial uses and medicinal features [74]. It includes 220 accepted taxa distributed in Europe, Northwest Africa, Ethiopia, Asia and southern Greenland [75,76,77]. The center of diversity of the genus is the Mediterranean region, where the typical endemism grows especially in the west sector (Iberian peninsula, Northwest Africa) [78]. In Italy, 20 species of *Th.* are reported, with 5 endemims of the southern regions (*Th. paronychioides* Čelak., *Th. picentinus* (Lacaita) Bartolucci, *Th. praecox* Opiz subsp. *parvulus* (Lojac.) Bartolucci, Peruzzi & N.G. Passal., *Th. richardii* Pers. subsp. *nitidus* (Guss.) Jalas, and *Th. spinulosus* Ten.) [26], where they occur mainly in rocky habitats, from hills to mountain tops. *Th. spinulosus* is reported in Campania, Apulia, Basilicata, Calabria and Sicily, while its presence is uncertain in Molise, no longer recorded in Latium, and reported by mistake in Abruzzo [26]. It grows in arid stony slopes, breached, clearings of deciduous oaks, from sea level up to about 1100 m of altitude and flowering in May–June. In Apulia, it is widespread in the perennial grasslands, especially in the pseudo-steppes of *Stipa austroitalica* Martinovský [29,54,79], which is considered is habitat (Eastern sub-Mediterranean dry grasslands (*Scorzoneretalia villosae*)” (code 62A0)) under Directive 92/43 EEC [64,65], while it is very rare in typically rocky vegetation [54].

Many species of *Thymus* are popular in the traditional medicine of many countries and nations as a source of valuable crude drug, where they are used for acute and chronic bronchitis, and the leaf extracts are prescribed as expectorant and analgesic [80]. In medicine and perfumery, *Th. serpyllum* and *Th. vulgaris* are widely used [81,82], and in general, many species of this genus show antimicrobial, anti-inflammatory, antioxidant, cytotoxic, spasmolytic, and antinematodal activities. *Thymus linearis* Benth. is used in Kashmir regions for gastrointestinal problems and fever issues [83]. *Thymus capitatus* (L.) Hoffmanns & Link (=*Thymbra capitata* (L.) Cav.) were used in Southern Italy to make fumigations in the treatment of colds together with dried figs and chestnuts [84]. Traditionally, the plants of the genus *Th.* are used as a source of EOs and are promising in pest management and control of harmful insects, especially thanks to thymol and carvacrol content, as well as citral, geraniol and nerolidol [85,86,87]. *Th.* still need to be explored as supplementary sources of botanical insecticides, to face the growing concerns arising from the use of chemical pesticides [88,89,90], with special reference to insecticide resistance [91]. *Th. spinulosus* is a less explored taxon for both phytochemistry and biological activities [92], and there are few works in the literature detailing information on its use in the kitchen such as the preparation called “sanguinaccio” in the Campania region, based on pig blood with dried fruit, sultanas and cooked wine [93]. The Sicilian data, obtained from four populations located above 1000 m of altitude, but in different soil conditions (siliceous and calcareous), gave oils with different composition, 62 components in total, which is inconsistent with the previous chemical composition of EOs described in the literature for this endemic taxon, suggesting a new chemotype, characterized by myrcene-limonene among monoterpenes and γ-muurolene, caryophyllene and germacra-1(10),4-dien-6-ol among sesquiterpenes as the main constituents [87].

The macroclimate in the two eastern sites (Ts1, Ts2) is Mediterranean, while in the Gargano, it is colder and temperate (Ts3). The bioclimate is Oceanic-semicontinental transition and subhumid Ombrotype in the two sunniest sites placed at higher altitudes (Ts2, Ts3), and Dry Mediterranean Oceanic type in cooler expositions, as observed at San Basilio (Ts1). The Gargano site differs from the other two sites also due to its geolithological characteristics, more recent (Pleistocene) for colluvial, terraced alluvial, fluviolacustrine and fluvioglacial deposits (Ts3) compared to those of the upper Cretaceous corresponding to skeletal limestones of neritic and carbonate platform facies (Ts1, Ts2). Additionally, the geopedology is different, with Tyrrhenian carbonate reliefs and material defined by calcareous sedimentary rocks on Gargano (Ts3), and Hilly reliefs with undifferentiated tertiary sedimentary rocks in the other two sites (Ts1, Ts2). This means that the three sites only have the same macroclimate, but the Gargano site (Ts3) differs from the other two due to different bioclimate, geology, and pedology characteristics, and partially different plant communities.

The phytosociological data (Table 7) show within the same alliance *Hippocrepido glaucae-Stipion austroitalicae*, there are two different plant associations, *Acino suaveolentis-Stipetum austroitalicae* (Ts1, Ts2) and *Sideritido syriacae-Stipetum austroitalicae* (Ts3). At S. Egidio (Ts3), in a good environmental condition, in the absence of human disturbances, with 626 m altitude, 12° slope, a medium level of stoniness (10%) and a high level of rockiness (45%), and 85% coverage of 64 plant species, we observed within the *Sideritido syriacae-Stipetum austroitalicae* association identified by *Sideritis italica*, a richness of species of *Hippocrepido glaucae-Stipion austroitalicae*, *Scorzoneretalia villosae* and *Festuco-Brometea* communities, with a good coverage of *Stipa austroitalica* subsp. *austroitalica*, *Scorzonera villosa* subsp. *columnae*, both endemics, and *Phleum hirsutum* subsp. *ambiguum*, in addition to other species such as *Petrorhagia saxifraga* subsp. *gasparrinii*, *Hippocrepis glauca*, *Teucrium capitatum* subsp. *capitatum*, *Convolvulus cantabrica*, *Koeleria splendens*, and *Eryngium amethystinum*. Some transgressive species of *Helianthemetea guttati* Rivas Goday & Rivas-Mart. 1963 and *Lygeo sparti-Stipetea tenacissimae* Rivas-Mart. 1978 classes show natural catenal contact with these other plant communities. In the two easternmost sites (Ts1, Ts2), always in natural conditions, but at different topographic conditions, such as altitudes (Ts1 = 274 m, Ts2 = 561 m), exposition (N–NE, S–SW) slope degree (2° and 10°), level of stoniness (5% and 20%) and rockiness (60% and 40%), we detected the same *Acino suaveolentis-Stipetum austroitalicae* association, characterized by three species, two of which are endemic and present at both sites (*Thymus spinulosus* and *Euphorbia nicaeensis* subsp. *japygica*), while *Clinopodium suaveolens* was present only at Scannapecora (Ts2). The taxa detected are comparable to those of Gargano (Ts3), except for the characteristics of the Festuco-Brometea class, which are less represented at both sites, explained by the lower plant biodiversity (Ts1 = 38 taxa, Ts2 = 44 taxa) than Gargano (Ts3 = 64 taxa), and only for Scannapecora (Ts2), also with good coverage of Ononido-Rosmarinetea transgressive species, which this plant association makes a mosaic.

The GC–MS results showed that EO composition consists of a total of 27 identified compounds for all three sites (Table 6). The environmental differences are reflected only partially in the chemical composition of EOs. Only low differences were observed on the abundance and patterns among the three sites, since they share 25 compounds; only 1 (Hotrienol) was exclusive to Ts1 (Hotrienol) and 1 to Ts2 (Caryophyllene oxide). The most interesting phytochemicals, having the highest presence in all samples, were thymol, p-Cymene and β-Ocimene. In particular, at San Basilio (Ts1), in the same climatic condition as Altamura (Ts2), but with lower altitudes and different topographic, geological and geopedological conditions, 28 compounds were detected, with the most abundant ones similar to the Altamura (Ts2) site. Slight differences in percentages were observed: thymol (42.9% and 48.8%, respectively), followed by p-Cymene (17.9% and 17.5%) and β-Ocimene (15.4% and 11.7%). The other compounds did not exceed 3% and 4%.

At the Gargano (Ts3) site, in a different climatic, environmental and partly different plant community, 28 compounds were extracted, comparable anywhere with the results of the other two sites (Ts1 and Ts2): thymol (45.9%), p-Cymene (17.5%) and β-Ocimene (11.0%). The abovementioned data differ from the Sicilian populations [86], and highlight a different chemotype, probably due to the lower altitude compared to the Sicilian stations, which exceed 1000 m above sea level, and maybe for the different latitude, while it seems that the climate, the pedological and lithological characteristics of the soil and the vegetation do not play a relevant role. In any case, the qualitative difference seems to be particularly relevant, since Sicilian populations show a number of components almost double that of Apulia.

### 4.3. Syntaxonomical Scheme of Surveyed Vegetation

 *Festuco-Brometea* Br.-Bl. et Tx. ex Soó 1947
  *Scorzoneretalia villosae* Kovačević 1959
   *Hippocrepido glaucae-Stipion austroitalicae* Forte et Terzi 2005 in Forte, Perrino and Terzi 2005
    *Hippocrepido glaucae-Stipienion austroitalicae* Biondi and Galdenzi 2012
     *Acino suaveolentis-Stipetum austroitalicae* Forte et Terzi in Forte, Perrino et Terzi 2005
     *Sideritido syriacae-Stipetum austroitalicae* Biondi and Guerra 2008
 *Ononido-Rosmarinetea* Br.-Bl. in A. Bolòs y Vayreda 1950
   *Cisto-Micromerietalia julianae* Oberd. 1954
    *Cisto cretici-Ericion manipuliflorae* Horvatić 1958
     *Phagnalo saxatilii-Saturejetum cuneifoliae* Biondi and Guerra 2008

## 5. Conclusions

The composition of *Satureaja cuneifolia* EOs is only partially affected by differences in environmental conditions, specifically for the geological, ecological, vegetational and pedological aspects. The soil in the two examined sites is rich in macro- and less rich in microelements, with differences in types and percentages. In particular, the microelements have similar concentrations, except for Zn and Mn. Among the total of 36 compounds detected and identified in the EOs extracted from the analyzed samples at both sites (Difesa di Malta—Sc1; San Basilio—Sc2), the most abundant compounds, often with strong differences between sites, were α-Terpineol, trans-3-caren-2-ol, and Borneol. At Difesa di Malta (Sc1), in more disturbed conditions and in an agriculture context, with a low plant biodiversity referable to an unrepresentative aspect of *Phagnalo saxatilii-Saturejetum cuneifoliae*, to a significant decrease in trans-3-caren-2-ol and borneol, there is a significant increase in α-Terpineol, compared to the better naturalness of San Basilio. Clearly, this means that the environment may play a strong role.

The composition of the *S. cuneifolia* EOs found in the present research does not match with those of previous studies conducted in the Mediterranean area [47,67,69], which showed different chemotypes also among themselves. In Croatia, the chemical composition shows a high percentage of carvacrol (17.7%), γ-terpinene (14.8%), p-cymene (9.8%), linalool (6.6%) and limonene (6.2%) [94]. In another work conducted in Salento (Apulia), but in different areas than the present one, there was a prevalence of linalool (9.6–32.7%), borneol (12.9– 24.0%) and α-pinene (9.5–11.7%) [70]. Recently, in Montenegro, it has been observed that the main constituents were linalool (20.3%), trans-(E)- caryophyllene (6.1%), germacrene D (5.8%), nerolidol (5.2%) and spathulenol (5.0%) [47]. Again, this confirms an environmental effect.

The climatic and geological factors, soil properties, and topographical and vegetational characteristics do not seem to play a crucial role on the chemical composition in EOs of *Thymus spinulosus* in Apulia, as observed in previous studies conducted in a Sicilian population where the environmental conditions and the different soil types affect the composition of EOs. The results of research conducted in Apulia indicate a total of 35 compounds for the three investigated sites, with the highest presence in all samples of thymol, p-Cymene and β-Ocimene, always with comparable percentages, unlike the Sicilian populations, with 62 components in four sites, geographically close and located at comparable altitudes, with variable values of the main components: myrcene (1.0–15.7%), limonene (2.3–13.2%), γ-muurolene (7.3–15.9%), caryophyllene (8.3–11.1%), and germacra-1(10),4-dien-6-ol (1.3–11.3%). 

Our results may highlight the following responses in the chemical composition of EOs of *Th. spinulosus*: (a) the Sicilian and Apulian populations are two different chemotypes and the Apulian ones are more stable in composition, regardless of environmental, climatic and vegetational factors; (b) latitude and altitude could be diagnostic factors, even if it would be interesting to complete the picture with other Italian populations such as those of Calabria; (c) it is confirmed that the genus *Th.* has several chemotypes. Furthermore, some studies underscore that other factors are also implicated in determining the EO composition of *Th.*, such as genetic and reproductive characteristics [94], the ecological functions of the EOs, e.g., protection from herbivores, interaction with microorganisms in the decomposition process, patterns of vegetation through allelopathic action [95], the physiological stage of the plant [87,96], and the negative impacts of herbicides [97].

Finally, adopting specific measures for selective management of the shrub vegetation is recommended, including those identified here, with a high concentration of species rich in EO, especially in the Mediterranean climate, in order to contribute to a reduction in the risk of fire [98] and safeguard the endemic and threatened flora, some of them still without studies of their chemical properties.

As general remarks, for both studied species, we may stress, once more, that the high bioactivity of *Th. spinulosus* indicates it has promising potential in organic agriculture, since it may provide thymol as a possible natural agent against phytopathogenic microorganisms; the richness of α-Pinene (insecticidal) in *S. cuneifolia* also makes the species a potential plant as natural pesticide in organic agriculture.

## Figures and Tables

**Table 1:** Phytoclimate at the investigated sites of *S. cuneifolia* (*Sc*) and *Th. spinulosus* (*Ts*).

Site (IC)	Macroclimate	Bioclimate	Ombrotype	Italian Distribution
*S. Egidio* *(Ts3)*	Temperate	Oceanic-semicontinental transition	Subhumid	Hilly areas of Apennines with Adriatic exposure
*Scannapecora (Ts2)*	Mediterranean			Coastal areas of the middle Adriatic, of the inland plains of the pre-Apennines and of Sicily
*San Basilio (Sc2, Ts1)*		Oceanic		Areas of the middle and lower Adriatic, the Ionian and the major islands; moderate presence also in the middle and upper Tyrrhenian regions
*Difesa di Malta (Sc1)*		Mediterranean oceanic	Dry	

**Table 2:** Geopedology, geolithology and ecopedology in the investigated sites of *S. cuneifolia* (*Sc*) and *Th. spinulosus* (*Ts*).

Site (IC)	Geo-Pedology and Geolithology	Ecopedology
*S. Egidio* *(Ts3)*	Colluvial, terraced alluvial, fluviolacustrine and fluvioglacial deposits (Pleistocene)	Tyrrhenian carbonate reliefs with material defined by calcareous sedimentary rocks and climate from Oceanic to Suboceanic Mediterranean partially mountainous
*Scannapecora (Ts2)*	Skeletal limestones of neritic and carbonate platform facies (Upper Cretaceous)	
*San Basilio (Sc2, Ts1)*		
*Difesa di Malta (Sc1)*	Terrigenous-skeletal limestones such as “Panchina” (Pleistocene)	Hilly reliefs with undifferentiated tertiary sedimentary rocks and sub-continental Mediterranean to continental Mediterranean climate

**Table 3:** Location, date retrieved, and geographical position in the investigated sites of *S. cuneifolia* (*Sc*) and *Th. spinulosus* (*Ts*).

IC	Taxon	Location	Date Retrieved	Geographic Position (WGS84)
Sc1	*Satureja cuneifolia* Ten.	Difesa di Malta (Fasano–Brindisi)	03.06.20	40.7986 N 17.4946 E
Sc2		San Basilio (Mottola–Taranto)	26.05.20	40.6877 N 16.9694 E
Ts1	*Thymus spinulosus* Ten.	San Basilio (Mottola–Taranto)	05.06.20	40.6877 N 16.9693 E
Ts2		Scannapecora (Altamura–Bari)	03.06.20	40.9051 N 16.4422 E
Ts3		S. Egidio (S. Giovanni Rotondo–Foggia)	12.06.20	41.7132 N 15.7658 E

**Table 4:** *Phagnalo saxatilii-Saturejetum cuneifoliae* Biondi & Guerra 2008.

*Identification Code*		Sc1	Sc2
Altitude (m a. s. l.)	endemic	49	274
Aspect		NE	N
Slope (°)		3	4
Relevé area (m^2^)		20	25
Stoniness (%)		20	10
Rockiness (%)		60	50
Cover total (%)		80	70
Number of species		23	41
Number of individuals collected for laboratory analysis		15	50
**Geolithology**		§	§§
**Ecopedology**		†	††
**Macroclimate**		≠	≠
**Bioclimate**		◊	◊◊
**Ombrotype**		#	##
Other species		10	10
**Characteristics association *Phagnalo saxatilii-Saturejetum cuneifoliae***			
*Satureja cuneifolia* Ten.		3	3
*Phagnalon saxatile* (L.) Cass.		+	-
**Characteristics Alliance *Cisto cretici-Ericion manipuliflorae*, Order *Cisto-Micromerietalia julianae*, Class *Ononido-Rosmarinetea***			
*Helianthemum jonium* Lacaita & Grosser		1	1
*Micromeria graeca* (L.) Benth. ex Rchb.		1	+
*Asphodeline lutea* (L.) Rchb.		-	1
**Characteristics Alliance *Hippocrepido glaucae-Stipion austroitalicae*, Order *Scorzoneretalia villosae***			
*Convolvulus cantabrica* L.		-	2
*Euphorbia nicaeensis* All. subsp. *japygica* (Ten.) Arcang.		-	2
*Teucrium capitatum* L. subsp. *capitatum*		-	2
*Petrorhagia saxifraga* (L.) Link subsp. *gasparrinii* (Guss.) Greuter & Burdet		-	1
*Eryngium amethystinum* L.		-	+
*Linum tommasinii* (Rchb.) Nyman		-	+
*Phleum hirsutum* Honck. subsp. *ambiguum* (Ten.) Cif. & Giacom.		-	+
*Scorzonera villosa* Scop. subsp. *columnae* (Guss.) Nyman	E	-	+
*Thesium humile* Vahl		-	+
*Thymus spinulosus* Ten.	E	-	+
**Characteristics Class *Festuco-Brometea***			
*Centaurea deusta* Ten.		-	2
*Anacamptis morio* (L.) R.M. Bateman, Pridgeon & M.W.Chase		-	+
*Galium corrudifolium* Vill.		-	+
*Poterium sanguisorba* L.		-	+
**Transgressive Class *Helianthemetea guttati***			
*Stachys romana* (L.) E.H.L. Krause		1	1
*Trifolium scabrum* L.		1	1
*Lagurus ovatus* L. subsp. *ovatus*		+	1
*Hypochaeris achyrophorus* L.		+	+
*Alkanna tinctoria* Tausch subsp. *tinctoria*		-	2
*Catapodium rigidum* (L.) C.E. Hubb.		+	-
*Aegilops ovata* auct.		-	+
*Festuca danthonii* Asch. & Graebn. subsp. *danthonii*		-	+
*Medicago truncatula* Gaertn.		-	+
*Plantago coronopus* L.		-	+
*Stipellula capensis* (Thunb.) Röser & H.R. Hamasha		-	+
**Transgressive Class *Lygeo sparti-Stipetea tenacissimae***			
*Daucus carota* L. subsp. *carota*		1	+
*Asphodelus ramosus* L. subsp. *ramosus*		+	-
*Convolvulus elegantissimus* Mill.		+	-
*Pallenis spinosa* (L.) Cass. subsp. *spinosa*		+	-
*Ferula communis* L. subsp. *communis*		-	+
*Reichardia picroides* (L.) Roth		-	+
Legend E = Endemic species			
**Geopedology and Geolithology**			
§—Terrigenus-skeletal limestones like “Panchina” (Pleistocene)			
§§—Skeletal limestones of neritic and carbonate platform facies (Upper Cretaceous)			
§§§—Colluvial, terraced alluvial, fluviolacustrine and fluvioglacial deposits (Pleistocene)			
**Ecopedology**			
†—Hilly reliefs with undifferentiated tertiary sedimentary rocks and sub-continental Mediterranean to continental Mediterranean climate			
††—Tyrrhenian carbonate reliefs with material defined by calcareous sedimentary rocks and climate from Oceanic to Suboceanic Mediterranean, partially mountainous			
**Macroclimate**			
≠—Mediterranean			
≠≠—Temperate			
**Bioclimate**			
◊—Mediterranean Oceanic			
◊◊—Oceanic			
◊◊◊—Oceanic-semicontinental transition			
**Ombrotype**			
#—Dry ##—Subhumid			

**Table 5:** Physico-chemical characteristics of *S. cuneifolia* (*Sc*) and *Th. Spinulosus* (*Ts*) for each investigated soil site.

*Soil Parameters*	Unit	Sc1	Sc2	Ts1	Ts2	Ts3
Sand (2–0.05 mm)	g∙kg^−1^	307	576	783	200	161
Silt (0.05–0.002 mm)	g∙kg^−1^	649	383	193	759	787
Clay (<0.002 mm)	g∙kg^−1^	44	41	23	41	51
Texture Class (USDA)		Silt loam	Sandy loam	Loam sand	Silt loam	Silt loam
Total Carbonate	g∙kg^−1^	306	698	965	19	<0.1
pH H_2_O	-	8.1	8.2	7.9	7.9	6.9
pH CaCl_2_	-	7.5	7.4	7.5	7.4	6.4
Electrical Conductivity (1:2)	dS∙m^−1^	0.2	0.2	0.3	0.2	0.2
Total Nitrogen	g∙kg^−1^	6.0	3.5	3.7	6.8	6.7
Available P_2_O_5_	mg∙kg^−1^	103	43	88	27	24
Organic Carbon	g∙kg^−1^	63	34	42	62	69
Organic Matter	g∙kg^−1^	109	59	72	107	119
C/N	-	10.5	10	11.2	9.2	10.3
Ca exchangeable	mg∙kg^−1^	9772	4842	4146	10,450	7711
K exchangeable	mg∙kg^−1^	253	93	87	515	855
Mg exchangeable	mg∙kg^−1^	240	131	105	231	368
Na exchangeable	mg∙kg^−1^	68	40	15	85	81
Available Fe	mg∙kg^−1^	13.1	7.8	8.9	20.3	31.9
Available Mn	mg∙kg^−1^	7.9	15.1	7.6	8.1	19.9
Available Zn	mg∙kg^−1^	2.5	20.5	8.9	1.6	3.0
Available Cu	mg∙kg^−1^	0.7	1.0	0.9	1.9	1.9
Available Ni	mg∙kg^−1^	0.27	0.27	0.14	0.16	0.21
Available Co	mg∙kg^−1^	0.03	0.06	0.03	0.03	0.11

**Table 6:** EO composition as area percentage of *S. cuneifolia* (*Sc*) and *Th. spinulosus* (*Ts*) with the identification method used. RT: retention time; RI: Kovats retention index; RI: experimental retention index; RI ref.: bibliographic retention index according to [32].

*Compound Name*	RT	RI	RI ref.	Sc1	Sc2	Ts1	Ts2	Ts3
*α*-Thujene	6.54	925	927	-	-	1.84	1.59	1.84
*α*-Pinene	6.74	932	936	36.8	38.82	0.95	0.73	0.83
Camphene	7.16	949	950	1.66	0.49	0.52	0.28	0.28
2,4(10)-Thujadiene	7.25	952	943	1.28	0.36	-	-	-
Sabinene	7.75	971	973	0.74	1.71	0.13	0.09	0.08
*β*-Pinene	7.89	977	977	0.36	0.38	0.87	0.72	0.94
*α*-Myrcene	8.16	987	989	0.84	1.43	1.33	1.1	0.83
2,3-Dehydro-1,8-cineole	8.19	989	989	1.78	0.99	-	-	-
3-Octanol	8.34	994	993	0.09	0.07	-	-	-
*α*-Phellandrene	8.63	1005	1004	0.81	0.71	0.18	0.14	0.18
α-Terpinene	8.94	1016	1017	0.14	0.11	2.24	1.84	2.9
*p*-Cymene	9.15	1023	1024	1.55	0.63	17.87	17.51	17.5
Limonene	9.29	1028	1029	5.08	6.39	0.51	0.41	1.43
*β*-Ocimene. (Z)	9.41	1033	1037	2.8	3.62	2.48	1.14	0.9
*β*-Ocimene. (E)	9.74	1044	1047	1.17	1.52	15.4	11.68	10.97
*γ*-Terpinene	10.10	1057	1060	0.25	0.19	-	-	-
Sabinene hydrate, *cis-*	10.43	1069	1066	0.12	0.1	0.45	0.49	0.52
Terpinolene	10.88	1085	1087	0.61	0.3	0.14	0.14	0.14
Linalool	11.25	1098	1099	6.35	6.36	1.83	2.41	2.9
Hotrienol	11.34	1101	1106	0.12	0.13	0.11	-	-
Thujone, cis	11.45	1104	1105	0.1	0.05	0.06	0.49	0.05
Chrysanthenone	11.89	1120	1124	0.31	0.1	-	-	-
*α*-Campholenal	12.00	1124	1124	1.05	0.33	0.05	0.05	0.19
Pinocarveol, *trans-*	12.47	1141	1140	1.78	0.78	-	-	-
*trans*-3-caren-2-ol	12.58	1146		4.1	1.9	-	-	-
Pinocarvone	13.05	1160	1160	0.33	0.12	-	-	-
Borneol	13.31	1170	1166	6.9	1.4	1	0.5	0.5
α-Terpineol	13.97	1193	1190	11.03	17.11	0.29	0.22	0.19
Carveol, *trans-*	14.60	1217	1217	0.92	0.35	-	-	-
Carvacrol, methyl ether	15.16	1238	1243	-	-	2.05	3.19	0.54
Bornyl acetate	16.42	1284	1283	0.21	0.07	-	-	-
Thymol	16.58	1289	1290	-	-	42.87	48.77	45.88
Carvacrol	16.77	1297	1300	-	-	0.94	0.62	2
Caryophyllene, (E)	20.01	1422	1420	0.07	0.08	2.07	1.22	2.22
Aromadendrene	20.48	1441	1440	0.21	0.35	-	-	-
*γ*-Curcumene	21.39	1478	1480	0.16	0.18	-	-	-
*α*-Bisabolene, (Z)	21.88	1497	1503	1.52	2.6	-	-	-
*γ*-Bisabolene	22.14	1509	1508	0.2	0.39	0.96	1.05	0.73
Caryophyllene oxide	23.75	1581	1580	3.25	2.44	-	0.14	-
Guaia-1(10),11-diene	24.81	1637	-	0.31	0.25	0.38	0.37	0.51
*Other components*				5	7.19	2.48	3.11	4.95

**Table 7:** *Hippocrepido glaucae-Stipion austroitalicae* Forte & Terzi in Forte, Perrino and Terzi 2005.

*Identification Code*		Ts1	Ts2	Ts3
Altitude (m a. s.l.)	e n d e m i c	274	561	626
Aspect		N-NE	S-SW	SE
Slope (°)		3	10	12
Relevé area (m^2^)		50	50	50
Stoniness (%)		5	20	10
Rockiness (%)		60	40	45
Cover total (%)		70	70	85
Number of species		38	44	64
Number of individuals collected for laboratory analysis		70	80	60
**Geolithology**		§§	§§	§§§
**Ecopedology**		††	††	††
**Macroclimate**		≠	≠	≠≠
**Bioclimate**		◊◊	◊◊◊	◊◊◊
**Ombrotype**		##	##	##
Other species		12	9	22
**Characteristics Association *Sideritido syriacae-Stipetum austroitalicae***				
*Sideritis italica* Mill.		-	-	2
**Characteristics Association *Acino suaveolentis-Stipetum austroitalicae***				
*Thymus spinulosus* Ten.	E	2	2	1
*Euphorbia nicaeensis* All. subsp. *japygica* (Ten.) Arcang.	E	1	+	-
*Clinopodium suaveolens* (Sm.) Kuntze		-	1	-
**Characteristics Alliance *Hippocrepido glaucae-Stipion austroitalicae***				
*Stipa austroitalica* Martinovský subsp. *austroitalica*	E	+	3	2
*Petrorhagia saxifraga* (L.) Link subsp. *gasparrinii* (Guss.) Greuter & Burdet		1	1	+
*Linum tommasinii* (Rchb.) Nyman		+	-	-
*Hippocrepis glauca* Ten.		-	-	+
**Characteristics Order *Scorzoneretalia villosae***				
*Teucrium capitatum* L. subsp. *capitatum*		1	1	1
*Thesium humile* Vahl		+	1	-
*Phleum hirsutum* Honck. subsp. *ambiguum* (Ten.) Cif. & Giacom.		1	-	3
*Convolvulus cantabrica* L.		1	-	+
*Scorzonera villosa* Scop. subsp. *columnae* (Guss.) Nyman	E	-	+	2
*Koeleria splendens* C. Presl	E	-	1	+
*Eryngium amethystinum* L.		-	+	1
*Satureja montana* L. subsp. *montana*		-	4	-
*Anthyllis vulneraria* L. subsp. *rubriflora* (DC.) Arcang.		-	1	-
*Teucrium capitatum* L. subsp. *capitatum*		1	1	1
**Characteristic Class *Festuco-Brometea***				
*Eryngium campestre* L.		+	2	-
*Centaurea deusta* Ten.		+	-	+
*Festuca circummediterranea* Patzke		-	2	+
*Poterium sanguisorba* L.		-	+	1
*Filipendula vulgaris* Moench		-	-	+
*Euphorbia spinosa* L.		-	-	1
*Helianthemum oelandicum* (L.) Dum. Cours. subsp. *incanum* (Willk.) G. López		-	-	1
*Melica ciliata* L. subsp. *ciliata*		-	-	+
*Potentilla calabra* Ten.	E	-	-	+
*Trifolium arvense* L.		-	-	+
*Trifolium campestre* Schreb.		-	-	+
**Transgressive Class *Helianthemetea guttati***				
*Lagurus ovatus* L.		1	1	2
*Aegilops ovata* auct.		+	1	+
*Hypochaeris achyrophorus* L.		+	1	+
*Stachys romana* (L.) E.H.L. Krause		+	1	+
*Trifolium scabrum* L.		1	1	-
*Alkanna tinctoria* Tausch subsp. *tinctoria*		1	+	-
*Ononis reclinata* L.		+	+	-
*Stipellula capensis* (Thunb.) Röser & H.R. Hamasha		+	+	-
*Avena barbata* Pott ex Link		+	-	+
*Trifolium stellatum* L.		+	-	+
*Linum strictum* L.		-	+	1
*Brachypodium distachyon* (L.) P. Beauv.		-	+	+
*Helianthemum salicifolium* (L.) Mill.		1	-	-
*Onobrychis aequidentata* (Sm.) d’Urv.		-	+	-
*Briza maxima* L.		-	-	1
*Hippocrepis ciliata* Willd.		-	-	1
**Transgressive Class *Lygeo sparti-Stipetea tenacissimae***				
*Asphodelus ramosus* L. subsp. *ramosus*		+	2	2
*Reichardia picroides* (L.) Roth		1	-	+
*Daucus carota* L.		+	-	+
*Carlina corymbosa* L.		-	+	+
*Convolvulus elegantissimus* Mill.		-	+	+
*Petrosedum ochroleucum* (Chaix) Niederle		-	+	+
*Ferula communis* L. subsp. *communis*		+	-	-
*Plantago bellardii* All. subsp. *bellardii*		-	1	-
*Dactylis glomerata* L. subsp. *hispanica* (Roth) Nyman		-	-	+
*Pallenis spinosa* (L.) Cass. subsp. *spinosa*		-	-	+
*Phlomis herba-venti* L. subsp. *herba-venti*		-	-	+
**Transgressive Class *Ononido-Rosmarinetea***				
*Micromeria graeca* (L.) Benth. ex Rchb.		-	1	1
*Satureja cuneifolia* Ten.		-	3	-
*Helianthemum jonium* Lacaita & Grosser		-	2	-
*Rhamnus saxatilis* Jacq.		-	+	-
Legend E = Endemic species				
**Geopedology and Geolithology**				
§—Terrigenus-skeletal limestones like “Panchina” (Pleistocene)				
§§—Skeletal limestones of neritic and carbonate platform facies (Upper Cretaceous)				
§§§—Colluvial, terraced alluvial, fluviolacustrine and fluvioglacial deposits (Pleistocene)				
**Ecopedology**				
†—Hilly reliefs with undifferentiated tertiary sedimentary rocks and sub-continental Mediterranean to continental Mediterranean climate				
††—Tyrrhenian carbonate reliefs with material defined by calcareous sedimentary rocks and climate from Oceanic to Suboceanic Mediterranean, partially mountainous				
**Macroclimate**				
≠—Mediterranean				
≠≠—Temperate				
**Bioclimate**				
◊—Mediterranean Oceanic				
◊◊—Oceanic				
◊◊◊—Oceanic-semicontinental transition				
**Ombrotype**				
#—Dry ##—Subhumid				

## Data Availability

The data that support the findings of this study are available from the corresponding author upon reasonable request.
